# A novel model describing blood pressure profiles

**DOI:** 10.3389/fcvm.2025.1583046

**Published:** 2025-06-06

**Authors:** Sabina Schlottau, Willi Cawello, Stephanie Läer

**Affiliations:** Institute of Clinical Pharmacy and Pharmacotherapy, Heinrich Heine University Düsseldorf, Düsseldorf, Germany

**Keywords:** blood pressure, model, circadian rhythm, blood pressure profile, blood pressure variability, cuffless blood pressure measurement

## Abstract

**Introduction:**

Blood pressure follows a circadian rhythm and is influenced by various factors. Blood pressure rises in the morning and decreases at night. It is known that deviations from this pattern are associated with an increased cardiovascular risk. Therefore, it is important to analyze blood pressure profiles and blood pressure variability.

**Methods:**

A blood pressure model was developed based on data from cuffless blood pressure measurements of six healthy volunteers over a 14-day period. Exponential formulas were applied for the description of blood pressure curves (systolic and diastolic), which were used for a non-linear regression model in R.

**Results:**

All six subjects showed a circadian pattern in systolic and diastolic blood pressure over a 14-day period. Both the measured values and the predicted values show that each subject's blood pressure was lower at night than during the day. Differences emerged in the level of blood pressure, the fluctuation, and the sleep times, which revealed individual characteristics in the daily blood pressure curves.

**Discussion:**

This novel blood pressure model can be used to visualize blood pressure profiles for several days and enables the assessment of the intra- and inter-individual variability of blood pressure.

## 1 Introduction

Hypertension is the most common preventable risk factor for cardiovascular deaths worldwide (1, 2). In 2019 high blood pressure was responsible for 19% of all deaths worldwide (2–4). To reduce blood pressure and the risk of mortality, a comprehensive understanding of the blood pressure profile and its variables is required.

It is well known that blood pressure changes throughout the day, following a circadian rhythm. In healthy people, blood pressure is higher during the wake phase and falls during the sleep phase (5–7). The morning blood pressure surge, about one hour before waking up, is influenced by sympathetic activity and the release of cortisol in the early hours of the morning, while the drop in blood pressure at night is influenced by melatonin production (5, 7, 8). Both the night dip and the morning blood pressure surge can provide an indication of cardiovascular risk (9–11). There are also indications that increased variability with normal blood pressure averages can lead to an increased cardiovascular risk (12, 13).

**Figure 1:**
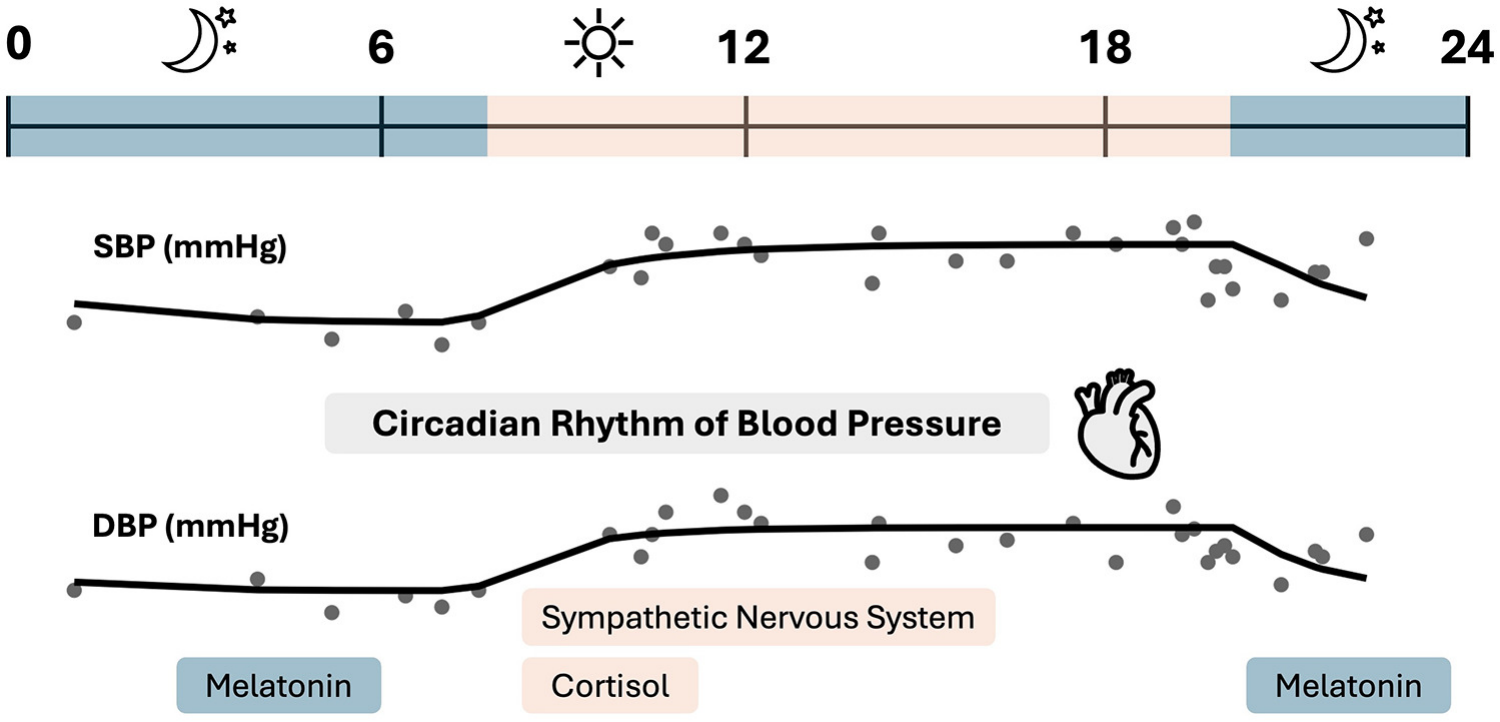
Blood pressure profile overview of one subject. The circadian blood pressure profile shows a lower blood pressure at night, which rises in the early morning hours. The nocturnal blood pressure drop is mainly influenced by melatonin, while the morning blood pressure surge is influenced by sympathetic activity and cortisol release. Created on the basis of (5, 8, 11, 14).

The blood pressure curve is influenced by intrinsic factors (e.g., Renin-angiotensin-aldosterone system, sympathetic activity, cortisol and melatonin release) (5, 8, 11, 14) as well as extrinsic factors (like lifestyle and environment) (15–17). A model can be used to visualize the changes in blood pressure, both for patients and for healthcare professionals. Figure 1 provides an overview of the blood pressure curve and its circadian rhythm.

A suitable blood pressure measurement method must be used to assess the course of blood pressure and metrics such as the night dip or blood pressure variability (13, 18). In the diagnosis of hypertension, ambulatory blood pressure monitoring (ABPM) is the gold standard (18–20). With ABPM blood pressure values are measured continuously (every 15–30 min) over a period of 24 h using an upper arm cuff. According to the ESC Guidelines the mean values of the systolic and diastolic blood pressures are used for assessment, more precisely the 24-hour mean value (should be <115/65 mmHg) as well as the day (<120/70 mmHg) and night (<110/60 mmHg) mean value. Although this measurement method provides continuous blood pressure values over a period of 24 h, it can affect the patient in everyday situations and during sleep due to the inflation of the cuff (18, 19).

To simplify blood pressure measurement over a longer period, cuffless blood pressure monitors have been developed (13, 21)*.* The blood pressure bracelet from Aktiia SA is one of the cuffless blood pressure monitors (22). The Aktiia blood pressure measurement system is a class IIa medical device (23). The bracelet enables resting blood pressure measurements over a period of more than 24 h (22). According to Sola et al. the Aktiia blood pressure bracelet is based on an optical measurement method, called photoplethysmography. Volume changes in a vascular system are measured by illuminating the skin of the wrist with a light-emitting diode and collecting the reflected light with a photodiode (22). This optical data received by the sensor is sent to the Aktiia app on the smartphone via a Bluetooth connection and from there to the Aktiia cloud. The blood pressure values calculated in the Aktiia cloud using the company's own algorithm are sent back to the app and are visible to the user (22). The blood pressure measurements are taken intermittently and automated, i.e., the measurements cannot be actively triggered by the user (23). So far, cuffless blood pressure monitors have not been recommended in the guidelines for either diagnosis or therapy support (19) but offer the potential to optimize the care of hypertension patients in the future.

In this study, blood pressure values obtained with the Aktiia bracelet were used to create a blood pressure model. Blood pressure models can be found in the literature and are mainly based on cosine methods (24, 25). Cosine models can be used to depict periodic recurring patterns. The blood pressure curve is periodic, but usually not symmetrical (24, 25). Madden et al. extended the simple cosine model and developed a multiple-component cosine random-effects model. Using their modelling approach, the authors also analyzed the variability between different individuals, but their focus was on quantifying the morning blood pressure surge rather than generating blood pressure profiles (25). The model of Head et al. followed a different approach, conducting a non-symmetrical double-logistic analysis of 24-h blood pressure profiles (26).

Exponential curves, on the other hand, occur in various natural processes (27, 28). This was a stimulus to develop the model according to exponential formulas. The model itself is a non-linear regression model developed and applied in R using procedure nls. R is an open-source programming language for statistical analyses (29). It enables individual adaptations, both in data analysis and visualization.

The main aim of this work was to describe and visualize blood pressure profiles over a period of two weeks using a newly developed blood pressure model and to provide a clearer impression of the observed daily values.

## 2 Material and methods

Data from a pilot project (approved by the ethics committee of medical faculty of Heinrich Heine University Duesseldorf, number: 2023–2409) conducted in 2023 with six subjects was used for the analysis. During the pilot project, six healthy adults (four students and two institute members) wore both a continuous glucose monitoring sensor (Abbott GmbH) and a cuffless blood pressure monitor (Aktiia SA) for a period of 14 days. The volunteers were 5 women and one man, with an average age of 25.5 years. The conditions for participation in the study were that the volunteers were healthy (without previous cardiovascular disease or diabetes mellitus) and had signed the informed consent form. During the 14 days, meals and activities were documented. A detailed description of the study design and the results for the four students can be found in a previous publication (30). One subject (9,905) also documented his sleeping times.

The focus of this work was to analyze the circadian rhythm of blood pressure in healthy adults. Firstly, a calculation function was created to describe the blood pressure. For the daytime blood pressure profile (blood pressure in the wake phase), it can be observed that blood pressure initially rises and then remains elevated for several hours. At bedtime, the blood pressure drops, then reaches a minimum until it rises again in the morning. Based on a visual inspection of the data, a baseline blood pressure (base) was assumed for the curve, from which the values rise in the morning.

### 2.1 Basic model

Due to the physiological nature of exponential processes, exponential functions were used to describe the rise and fall in blood pressure. The extent to which the blood pressure changes was defined as the parameter incr. The parameter k describes the blood pressure changes as a rate. The time t1 was defined as the starting point for wake time and t2 as bedtime. This represents the basic model formula:

It was considered whether it was the wake phase during the day (t1 < t < t2),

$$\left(EQ1)\;bp[i]=base+incr*(1-e^{-k*(t-t1)}\right)$$

the time at night after which people become inactive (bedtime, t > t2),

$$(EQ2)\;bp[i]=base+incr*(1-e^{-k*(t2-t1)})*e^{-k*(t-t2)}\;\;$$

(untilmidnight)

or the time at night before people become active (t < t1).

$$(EQ3)\;bp[i]=base+incr*(1-e^{-k*(t2-t1)})*e^{-k*(t+24-t2)}\;\;$$

(betweenmidnightandwakingup)

### 2.2 Extended model

Assuming that the physiological day does not range from midnight to midnight, but is limited by sleeping times, the basic model described above was extended with parameters from the previous day. The parameters base and incr up to t1 were taken from the previous day. The reason for this is the assignment of the sleep phase up to t1 to the previous day. The calculation formula (EQ3) was adjusted accordingly:

$$(EQ4)\;bp[i]=base\_prev+incr\_prev*(1-e^{-k*(t2-t1)})*e^{-k*(t+24-t2)}$$

base_prev and incr_prev are the parameters base and incr of the previous day.

The basic model formula and the extended formula were used for modelling with measured data of blood pressure, respectively. The model was developed and applied using procedure nls in R (version 4.4.2) (29).

Assuming that the base parameter represents a baseline, the minimum systolic or diastolic blood pressure was used as the starting parameter, respectively. As the parameter incr describes the extent of the change in blood pressure from the baseline, the difference between the minimum and maximum blood pressure was used as the starting parameter. After inspecting the data, 0.3 was selected as the starting parameter for k, 5 for t1 and 22 for t2. The port algorithm was used to set limits (lower = c[80 (systolic)/20 (diastolic), 0, 0.01, 1, 18], upper = c[200 (systolic)/120 (diastolic), 60, 2, 11, 25]). Another special feature is that the model was implemented in a double loop, i.e., the modelling was carried out in two coordinated steps. In the first loop, the blood pressure values were assigned to the subjects. In the second loop, a detailed analysis of the data for each individual day was carried out within each subject. The model reviewed the specific data and patterns related to the individual characteristics of the subjects for each day.

The data of all subjects were used simultaneously to obtain data on the study population as well as individual subjects. In addition to the predicted blood pressure values, the model results were the weighted residuals (residuals divided by the observed values in percent) and the parameter estimates (base, incr, k, t1, t2). The model was also used to estimate intra- and inter-individual variability. Inter-individual variability describes the differences between individuals and intra-individual variability describes the differences within an individual over time.

### 2.3 Statistics

Parameter median values were used to describe the differences, as the study population was small, and the values were widely spread. The range (maximum and minimum) was also included. To determine the intra-individual variability, the coefficient of variation of the parameters base and incr was calculated for each subject over the different days. To determine the inter-individual variability, the coefficient of variation of the parameters base and incr was calculated across all subjects.

During model development and to assess the fit of the model to the data, goodness-of-fit plots were created. These included plots of the measured blood pressure values compared to the predicted blood pressure values. Plots were also generated to assess the weighted residuals over time. Ideally, the weighted residuals should be randomly distributed around zero and not show any systematic patterns. These weighted residual plots were also used to compare the basic model with the extended model.

## 3 Results

The 14-day blood pressure measurement with a cuffless blood pressure monitor was completed by six young, healthy adults. The characteristics of the subjects are listed in Table 1. On average, 32 measurements per day were conducted. A more detailed list of the number of measurements can be found in Supplementary Table S1.

**Table 1:** Participant characteristics.

Characteristics	Mean (±SD), range
Age (Y)	25.5 (±2.7), 22–29
Sex, percentage of women (%)	83.3
BMI (kg/m^2^)	24.8 (±4.0), 19.8–30.8
Hypertensives (%)	0
Smokers (%)	0
Diabetic patients (%)	0

**Figure 2:**
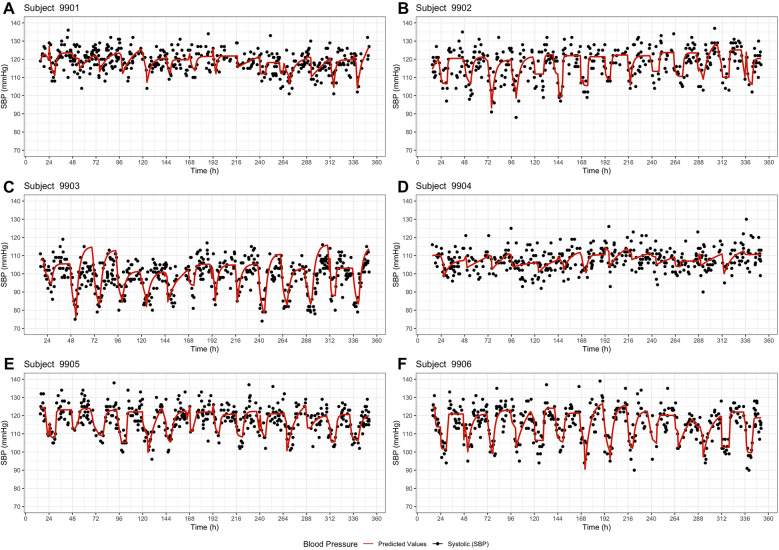
Basic model – systolic blood pressure profiles of the six subjects **(A–F)** over a period of 14 days. The dots represent the observed systolic blood pressure values, and the red line represents the model prediction.

**Figure 3:**
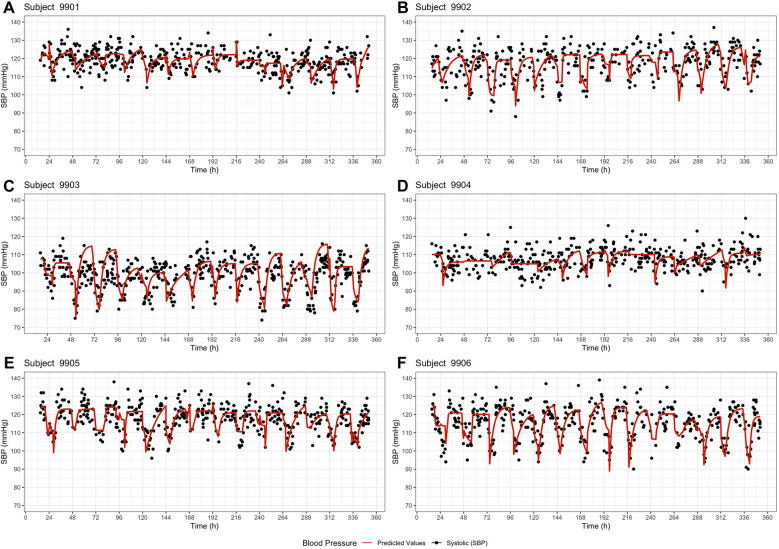
Extended model – systolic blood pressure profiles of the six subjects **(A–F)** over a period of 14 days. The dots represent the observed systolic blood pressure values, and the red line represents the model prediction including the parameters base and incr of the previous day.

All six subjects showed a circadian pattern in both systolic and diastolic (see Supplementary Material) blood pressure over a 14-day period. It was observed that blood pressure was lower at night than during the day (in the wake phase). Both the measured values and the predicted values show that each subject had an individual blood pressure curve and also a different range (Figure 2). The six subjects showed differences regarding the level of blood pressure, the fluctuation and the sleep times. This can be seen both in the graphical visualization (Figure 2, Figure 3) and in the parameter estimates (Tables 2, 3). Of particular interest for the blood pressure assessment are the parameters base and incr as they provide a picture of the blood pressure level and the extent to which the blood pressure fluctuates.

**Table 2:** Parameter estimates and inter-/intra-individual variability for systolic blood pressure (basic model).

Subject	Median with range (minimum—maximum)					Coefficient of variation (%)	
	base	incr	k	t1	t2	base	incr
9,901	112.0 (80.0–115.6)	13.9 (6.2–59.4)	0.25 (0.01–2.00)	4.30 (1.03–11.00)	24.43 (20.08–25.00)	8.0	75.7
9,902	106.2 (80.0–113.4)	15.0 (10.3–44.2)	0.81 (0.14–2.00)	6.18 (1.75–8.75)	22.83 (18.80–24.96)	8.2	47.1
9,903	82.1 (74.0–98.4)	22.0 (9.3–40.0)	0.30 (0.09–2.00)	5.00 (1.00–5.94)	22.47 (20.21–24.42)	8.5	43.4
9,904	103.7 (97.2–110.6)	10.9 (0.0–31.2)	0.07 (0.01–2.00)	3.53 (1.00–5.73)	23.85 (19.12–25.00)	3.3	64.0
9,905	107.6 (97.5–112.4)	15.1 (10.9–33.8)	0.65 (0.10–2.00)	5.14 (1.00–7.38)	21.38 (18.00–24.99)	4.3	40.0
9,906	101.7 (90.6–113.8)	20.8 (11.2–37.0)	0.40 (0.06–2.00)	5.35 (3.58–11.00)	22.37 (18.00–24.58)	5.5	30.3
Overall	104.0 (74.0–115.6)	15.6 (0.0–59.4)	0.33 (0.01–2.00)	4.83 (1.00–11.00)	22.99 (18.00–25.00)	10.3	52.8

**Table 3:** Parameter estimates and inter-/intra-individual variability for systolic blood pressure (extended model).

Subject	Median with range (minimum—maximum)					Coefficient of variation (%)	
	base	incr	k	t1	t2	base	incr
9,901	106.0 (80.0–121.8)	13.8 (1.0–43.0)	0.26 (0.08–2.00)	3.59 (1.00–11.00)	24.14 (21.88–25.00)	9.0	67.1
9,902	101.3 (80.0–117.9)	20.7 (3.6–44.2)	0.33 (0.10–2.00)	4.60 (1.00–6.85)	22.17 (18.18–24.58)	9.7	47.7
9,903	81.3 (74.0–98.4)	22.0 (9.3–40.0)	0.30 (0.07–1.22)	5.00 (1.00–5.60)	22.00 (19.31–25.00)	8.2	40.4
9,904	103.4 (91.7–110.1)	12.9 (0.0–54.8)	0.19 (0.01–1.81)	2.22 (1.00–5.88)	23.31 (20.98–25.00)	5.8	107.1
9,905	105.6 (80.0–117.1)	16.4 (7.0–41.7)	0.52 (0.07–2.00)	4.14 (1.00–9.88)	22.98 (20.09–24.91)	9.5	51.1
9,906	94.3 (82.7–120.1)	26.6 (0.0–36.6)	0.26 (0.12–2.00)	5.25 (1.63–11.00)	22.23 (18.00–24.03)	10.8	47.0
Overall	101.4 (74.0–121.8)	17.4 (0.0–54.8)	0.30 (0.01–2.00)	4.17 (1.00–11.00)	22.84 (18.00–25.00)	11.6	58.6

Analyzing the blood pressure of subject 9,906, both the basic model and the extended model demonstrated a regular rise and fall in blood pressure. The observed systolic blood pressure of 9,906 was in a range of 90–140 mmHg and the diastolic values were between 50 and 85 mmHg. The basic model predicted a base-range of 90.6–113.8 mmHg and an incr-range of 11.2–37.0 mmHg, while the extended model predicted a base-range of 82,7–120.1 mmHg and an incr-range of 0.0–36.6 mmHg. A median baseline blood pressure of 101.7 mmHg and a median incr of 20.8 mmHg (Table 2) indicates that the average systolic blood pressure during the day was around 123 mmHg and around 102 mmHg during the night. Subject 9,903 also showed highly fluctuating values, but in a lower range. The observed systolic blood pressure values ranged from 74 to 119 mmHg and the diastolic values from 41 to 73 mmHg. The model predicted median base values of 82.1 mmHg (basic) and 81.3 mmHg (extended), and median incr values of 22 mmHg (both models). In contrast, the subject 9,904 showed only low fluctuation. The median of the parameter incr was the lowest in 9,904 (10.9 mmHg basic/12.9 mmHg extended model). Furthermore, the basic model predicted a circadian pattern, while the extended model did not predict all night dips in 9,904. These differences in the parameters between the subjects suggest a high inter-individual variability. Considering the blood pressure of the individual subjects on the different days (Figures 2, 3), there are only slight differences. The coefficient of variation shows also that the fluctuations of the parameter incr are greater than for the parameter base, both inter- and intra-individually.

**Figure 4:**
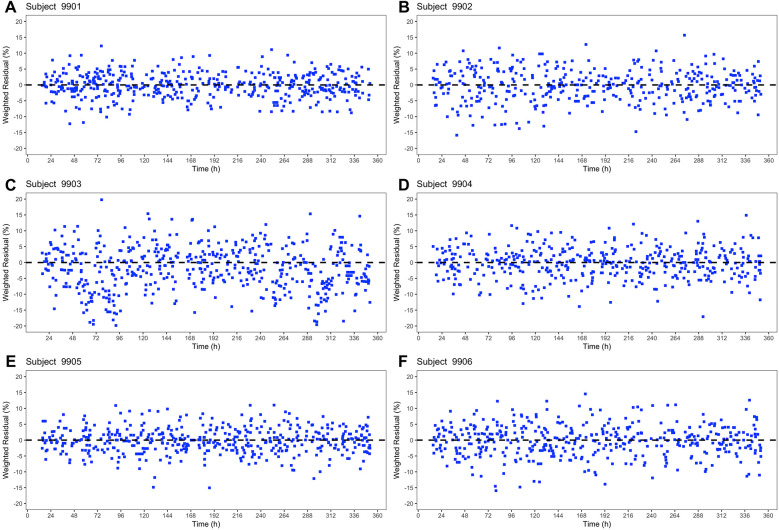
Basic Systolic Blood Pressure Model – Plot of the weighted residuals (%) for all six subjects **(A–F)** in the shape of blue squares against time (h).

**Figure 5:**
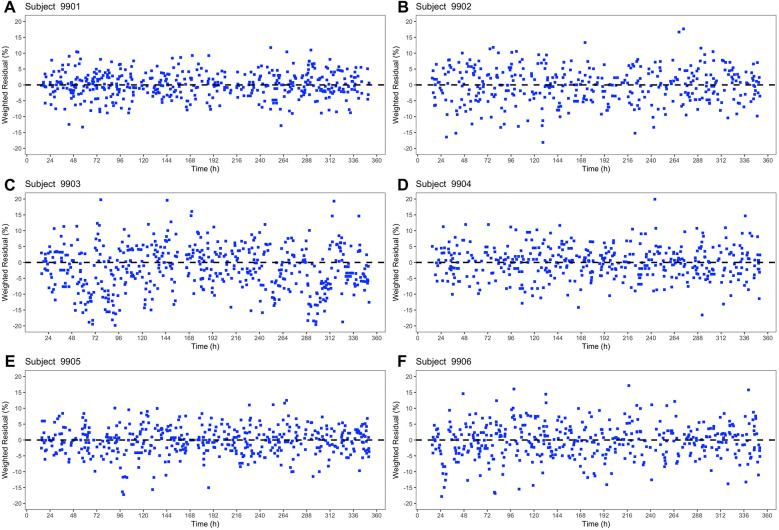
Extended Systolic Blood Pressure Model – Plot of the weighted residuals (%) for all six subjects **(A–F)** in the shape of blue squares against time (h).

The weighted residuals (%) for the six subjects in Figure 4 were evenly distributed over the 14-day period. The constant distribution over time shows that there is no time dependence of the weighted residuals. There were only minor differences between the basic model and the extended model (Figures 4, 5) in the weighted residuals. The scatter of the weighted residuals was the greatest in subject 9,903 and the lowest in 9,901 in both models. The weighted residuals showed no systematic trends or patterns, indicating a good model fit.

## 4 Discussion

This work demonstrates that it is possible to visualize and analyze blood pressure profiles over a 14-day period using a model and data from cuffless blood pressure monitoring. We have also shown that the model based on exponential formulas can be extended by including parameters from the previous day. The presented blood pressure model can be used to visualize blood pressure profiles of individual subjects, but also enables the assessment of the intra- and inter-individual variability of blood pressure. To our knowledge, this is the first blood pressure model that is based on values from a cuffless blood pressure monitor and uses exponential formulas.

Several blood pressure models have been published so far. The model by Head et al. considers the daytime and night-time blood pressure values and the transitions between those phases. The double logistic curves show parallels to our predicted curves, but their model does not consider blood pressure variability over time (26). More commonly used cosine models assume that blood pressure follows a symmetrical pattern and thus ignore the complexity of blood pressure profiles. For this reason, Madden et al. developed a multiple-component cosine random-effects model. This was used to quantify blood pressure variability (25). However, this model is also limited to 24 h. Parati et al. analyzed the benefits of 24-h blood pressure models. The authors concluded that most models can be useful in describing 24-h blood pressure profiles, but have a limited data set. Therefore, they may not reflect a subject's actual blood pressure profile (24). This is where our model makes a difference, because it is able to fit over several days. This allows an adjustment to the actual blood pressure variability in consideration of the individual days. Due to the small number of subjects, it is not yet possible to quantify the inter- and intra-variability precisely. The estimation based on the parameter medians and ranges as well as the coefficient of variation and the graphical representation reflect the expectation that the inter-individual differences are greater than the intra-individual differences. The greater variation of the parameter incr than the parameter base could indicate that incr is particularly influenced by different circumstances, whereas base could be a parameter that is specific to an individual. The extended model seems to give smoother transitions between day and night, but only if there is a pronounced night dip. Interestingly, one of the healthy subjects did not show a clear night dip. For this individual, the base model was able to describe its individual blood pressure values. In any case, further examination of other factors, such as sleeping patterns, which might influence the blood pressure should be regarded.

This work has several limitations. Data from a pilot project with only six participants was used. Therefore, the blood pressure model was only applied to these six subjects and inter-individual variability of model parameters could not be further evaluated. A larger study population could possibly provide further insights, where attention should be paid to an equal gender distribution. At this point it should be emphasized that the non-linear regression model used was adjusted individually for each subject. There is no model adjustment across all subjects. Therefore, it can be assumed that the validity of the model does not depend on the number of subjects included, but rather on the quality of the fit within the individual data sets. Nevertheless, the focus of the pilot project conducted in 2023 was on the effects of certain meals and activities on glycemic control with simultaneous blood pressure monitoring (30). The study protocol did not include explicit monitoring of the circadian rhythm at this stage, so documentation of bedtimes was not mandatory. Only one subject monitored their sleep times with an Apple Watch out of their own interest. Due to the limited comparability with the other data sets, the sleep data including sleep phases were not included in the analysis. It could be useful to place a greater focus on the exact daily routine in further studies and analyses. In addition, four of the six subjects were students, whose lifestyles probably do not reflect the average lifestyle of the population (e.g., in terms of sleep behavior and stress levels). Although age and body mass index (BMI) were considered in the data collection, the present analysis did not reveal any systematic correlations with the vital parameters recorded. Initial observations only indicated that the person with the lowest BMI also had the lowest blood pressure values, while no clear pattern could be identified in the remaining sample. Due to the small age range (22–29 years), no relevant differences were found here either. Therefore, these variables were not included in the model described.

Another limitation is the data collection itself. The Aktiia bracelet is a validated medical device and measures blood pressure automatically and unnoticed, but irregularly (23). In contrast to ABPM, measurements are not taken at a defined time interval. However, measurements can be taken in everyday life without major restrictions, even over several days or weeks. In addition, the measurements are taken in a resting position (23). Because of these characteristics of the Aktiia bracelet, it is not possible to create a complete and seamless blood pressure profile or to measure blood pressure during physical activity. Although physical activity is an important factor for cardiovascular risk assessment, this aspect could not yet be included in the model.

In summary, knowledge of a person's blood pressure profile over a period of more than 24 h can offer the opportunity to respond to individuals more personalized. A further and broader application of the blood pressure model would be desirable. In the future, simulations could be possible and co-variates could be identified. This would require further development of the model and a larger number of subjects. The authors are optimistic that further research could lead to implementation in healthcare.

## Data Availability

The raw data supporting the conclusions of this article will be made available by the authors, without undue reservation.
