# Supplementary material for: A novel model describing blood pressure profiles
Source: Front Cardiovasc Med. 2025 Jun 6;12:1583046. doi: 10.3389/fcvm.2025.1583046 (PMC12179137; doi:10.3389/fcvm.2025.1583046)
Supplement: Supplementary file 1 [file Datasheet1.pdf]

Supplementary Material

1 Supplementary Figures and Tables

1.1 Supplementary Figures

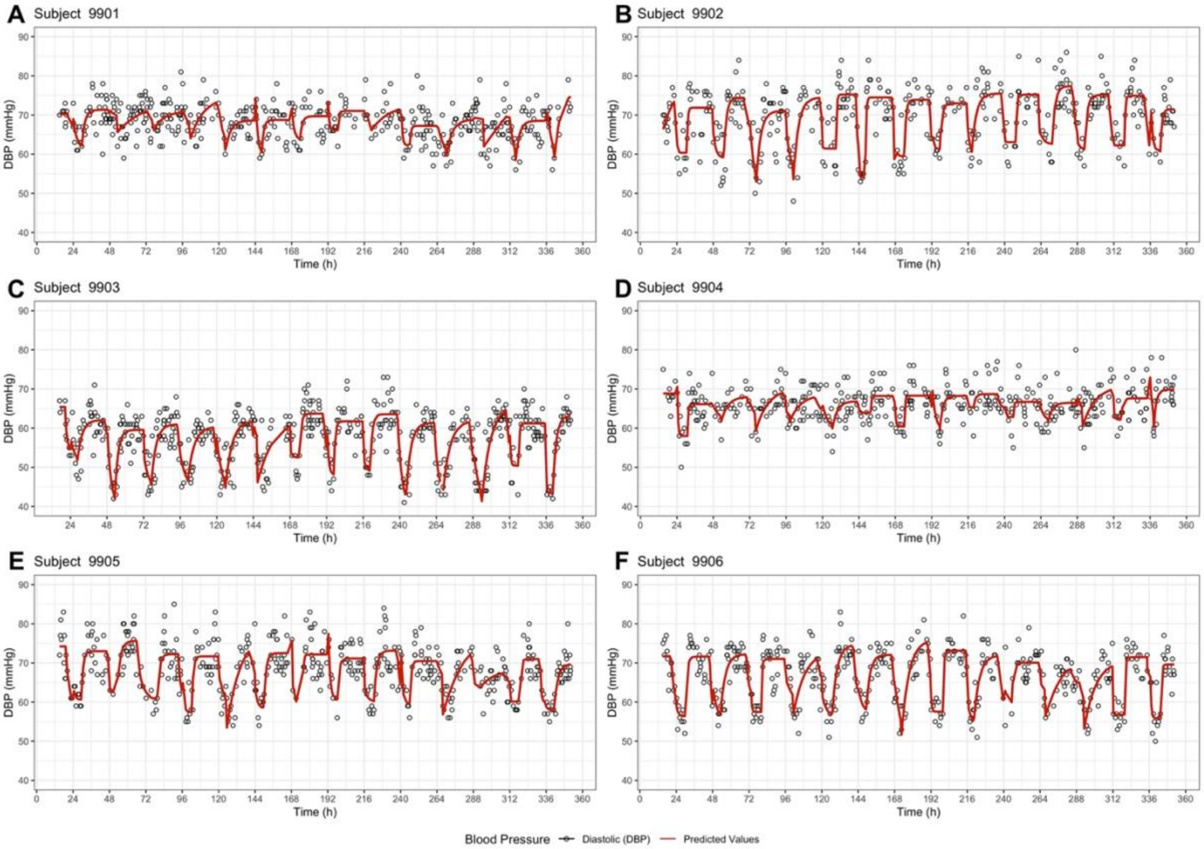

Supplementary Figure 1. Basic Model – Diastolic blood pressure profiles of the six participants (A-F) over a period of 14 days. The dots represent the observed diastolic blood pressure values, and the red line represents the model prediction.

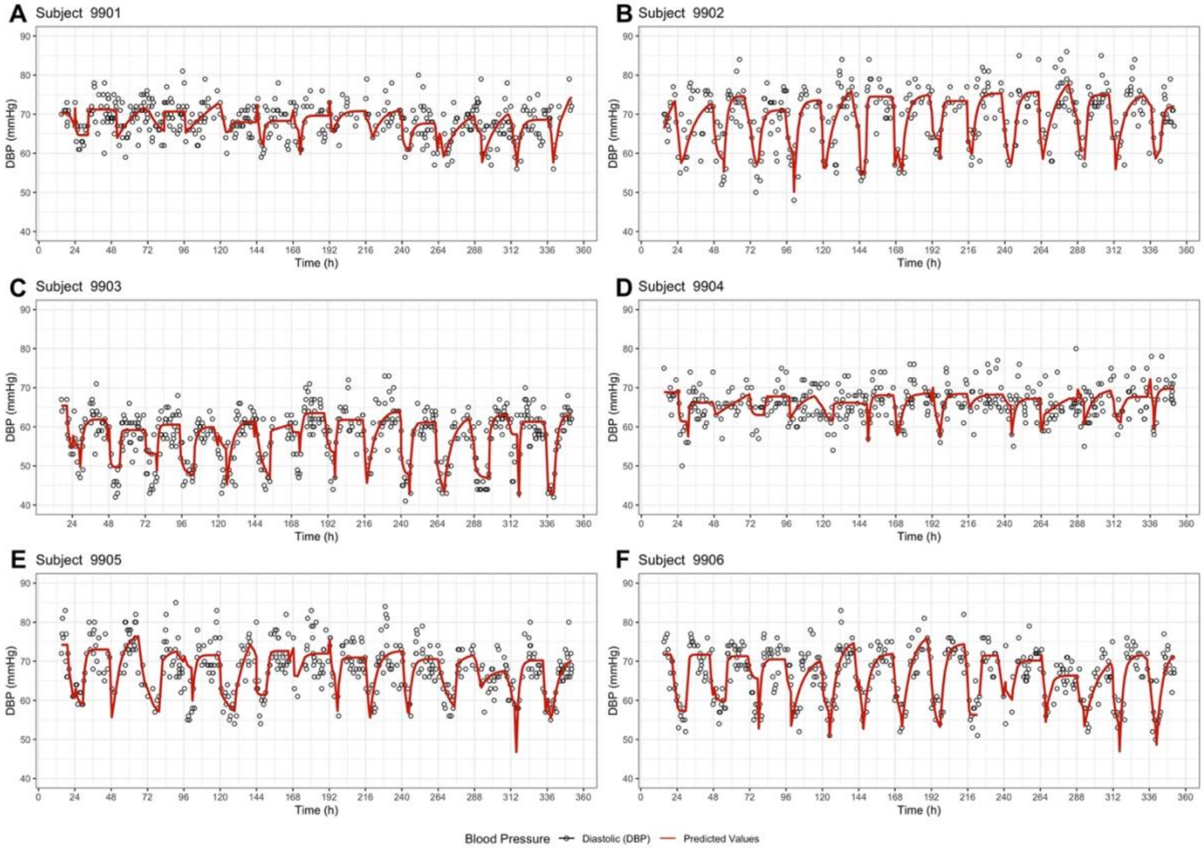

Supplementary Figure 2. Extended Model – Diastolic blood pressure profiles of the six participants (A-F) over a period of 14 days. The dots represent the observed diastolic blood pressure values, and the red line represents the model prediction including the parameters base and incr of the previous day.

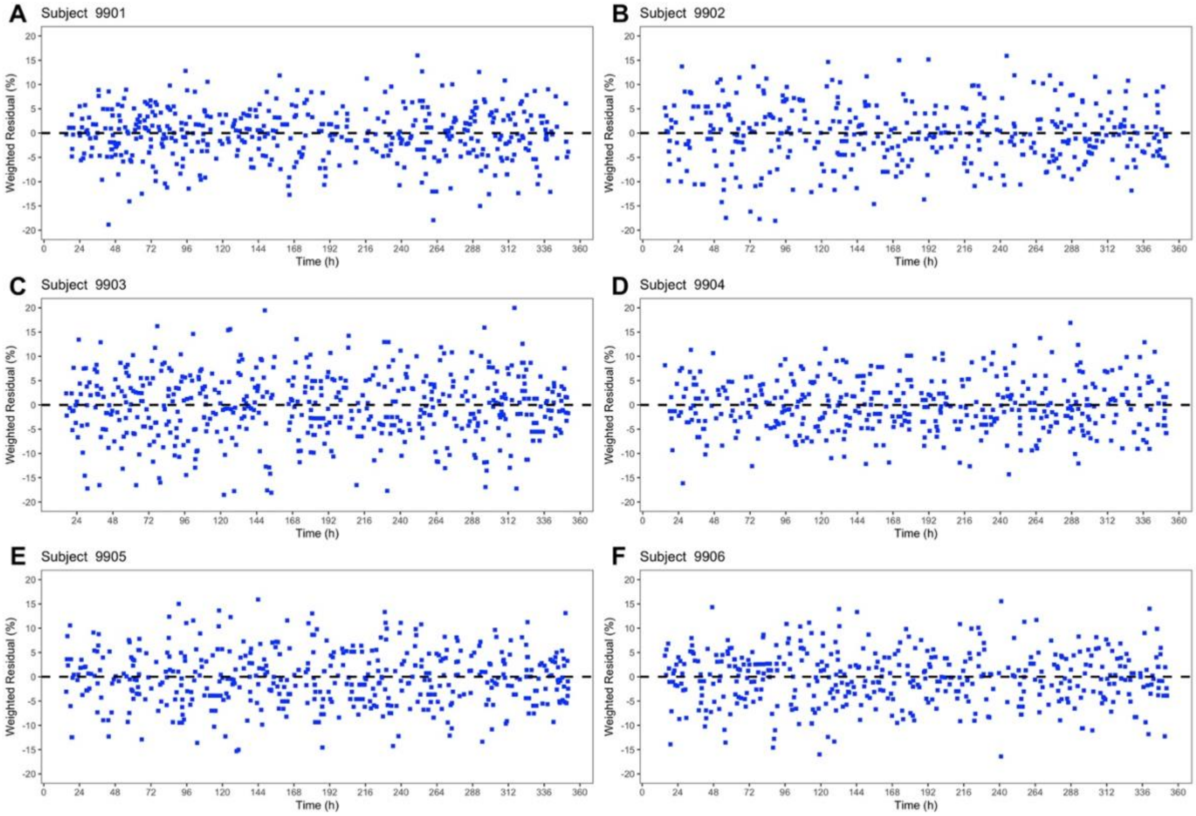

Supplementary Figure 3. Basic Diastolic Blood Pressure Model – Plot of the weighted residuals (%) in the shape of blue squares against time (h)

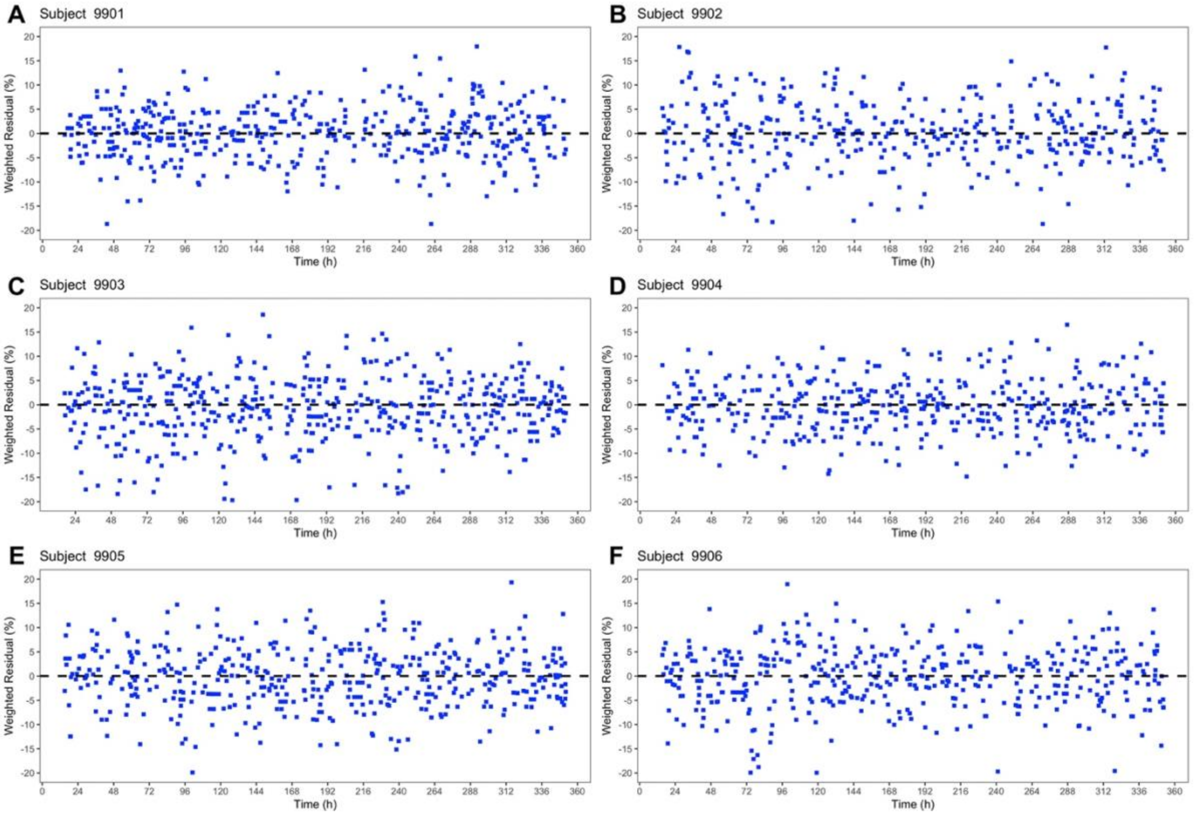

Supplementary Figure 4. Extended Diastolic Blood Pressure Model – Plot of the weighted residuals (%) in the shape of blue squares against time (h)

1.2 Supplementary Tables

Supplementary Table 1. Number of measurements

Subject	Number of measurements/ 14 days	Average of measurements/ day
9901	469	34
9902	398	28
9903	531	38
9904	431	31
9905	461	33
9906	438	31
Mean overall	455	32

Supplementary Table 2. Parameter Estimates and Inter-/intra-individual Variability for Diastolic Blood Pressure (Basic Model)

Subject	Median with range (minimum - maximum)					Coefficient of Variation (%)	
	base	incr	k	t1	t2	base	incr
9901	62.3 (56.3-66.1)	8.4 (4.9-55.7)	0.29 (0.01-1.5)	5.17 (3.78-11.00)	24.10 (21.45-25.00)	4.7	109.5
9902	60.4 (26.2-63.1)	14.5 (10.8-52.9)	0.91 (0.11-2.00)	5.97 (1.75-8.75)	22.55 (19.96-24.62)	16.4	58.6
9903	44.4 (36.1-54.3)	16.6 (10.8-30.0)	0.34 (0.10-2.00)	4.60 (1.00-5.98)	22.52 (18.00-24.31)	12.3	32.1
9904	60.9 (58.0-65.3)	7.5 (3.4-11.9)	0.62 (0.12-2.00)	4.88 (1.00-7.71)	23.37 (18.85-24.82)	3.3	35.9
9905	60.1 (51.6-62.3)	12.4 (6.8-26.1)	0.70 (0.07-2.00)	5.70 (1.00-7.45)	21.75 (18.00-24.92)	4.5	35.6
9906	56.5 (48.1-60.0)	15.6 (10.1-28.0)	0.42 (0.08-2.00)	5.25 (1.00-8.00)	22.12 (19.36-24.84)	5.2	24.1
Overall	59.3 (26.2-66.1)	12.6 (3.4-55.7)	0.53 (0.01-2.00)	5.05 (1.00-11.00)	22.58 (18.00-25.00)	13.1	59.1

Supplementary Table 3. Parameter Estimates and Inter-/intra-individual Variability for Diastolic Blood Pressure (Extended Model)

Subject	Median with range (minimum - maximum)					Coefficient of Variation (%)	
	base	incr	k	t1	t2	base	incr
9901	61.2 (52.1-70.7)	11.7 (0.0-24.5)	0.30 (0.02-2.00)	3.43 (1.10-11.00)	23.53 (21.92-25.00)	9.9	66.2
9902	55.2 (26.2-63.6)	19.5 (11.4-52.9)	0.43 (0.11-2.00)	4.14 (1.00-6.48)	22.64 (18.00-24.42)	19.7	45.2
9903	47.8 (20.1-58.6)	12.9 (1.9-41.7)	0.60 (0.18-2.00)	5.45 (1.00-10.39)	22.64 (19.97-24.60)	22.2	68.2
9904	61.4 (44.0-67.8)	9.8 (0.0-26.6)	0.46 (0.01-2.00)	3.90 (1.00-9.29)	23.12 (18.57-24.99)	11.2	74.2
9905	55.0 (39.4-68.5)	17.6 (3.8-31.8)	0.36 (0.10-1.07)	4.39 (1.00-11.00)	21.77 (18.00-24.88)	16.0	50.7
9906	52.0 (34.1-65.5)	18.2 (5.8-37.4)	0.41 (0.11-1.92)	4.48 (1.00-7.92)	22.68 (20.53-25.00)	13.6	38.1
Overall	55.1 (20.1-70.7)	16.2 (0.0-52.9)	0.44 (0.01-2)	4.27 (1.00-11.00)	22.70 (18.00-25.00)	17.5	60.8
